# Flowtigs: Safety in flow decompositions for assembly graphs

**DOI:** 10.1016/j.isci.2024.111208

**Published:** 2024-10-25

**Authors:** Francisco Sena, Eliel Ingervo, Shahbaz Khan, Andrey Prjibelski, Sebastian Schmidt, Alexandru Tomescu

**Affiliations:** 1 University of Helsinki, Helsinki, Finland; 2 Indian Institute of Technology Roorkee, Roorkee, India

**Keywords:** Microbial genomics, Biocomputational method, Classification of bioinformatical subject, Genomic analysis

## Abstract

A *decomposition* of a network flow is a set of weighted walks whose superposition equals the flow. In this article, we give a simple and linear-time-verifiable complete characterization (*flowtigs*) of walks that are *safe* in such general flow decompositions, i.e., that are subwalks of any possible flow decomposition. We provide an *O*(*mn*)-time algorithm that identifies all maximal flowtigs and represents them inside a compact structure. On the practical side, we study flowtigs in the use-case of metagenomic assembly. By using the species abundances as flow values of the metagenomic assembly graph, we can model the possible assembly solutions as flow decompositions into weighted closed walks. On simulated data, compared to reporting unitigs or maximal safe walks based only on the graph structure, reporting flowtigs results in a notably more contiguous assembly. On real data, we frame flowtigs as a heuristic and provide an algorithm that is guided by this heuristic.

## Highlights

• We propose flowtigs, a generalization of unitigs that take abundances into account
• Flowtigs are efficiently computable on graphs built from error-free and real data
• Experiments on metagenomic sequencing data show notable improvements over unitigs

## Graphical abstract

**Figure:**
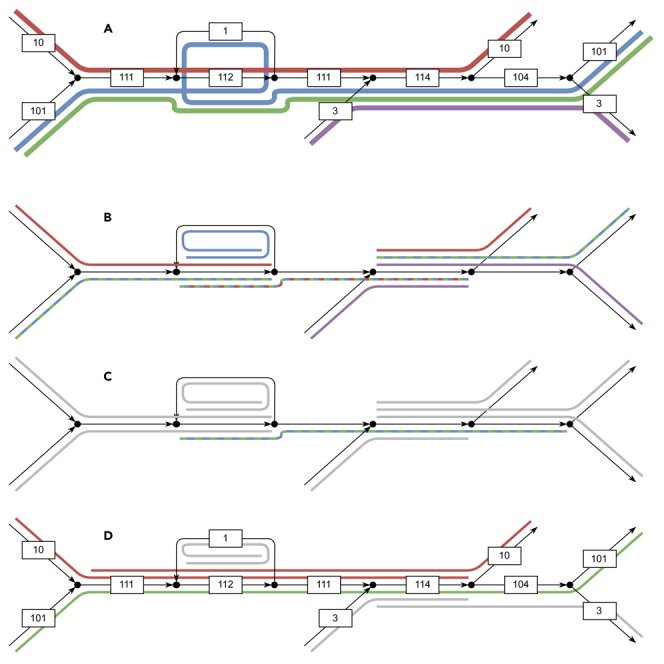

## Introduction

Network flows are a useful model in assembly problems since they not only take into account the graph structure but also an abundance of information. In practice, this information is often readily available. For example, in genome or metagenomic assembly, the nodes or the arcs of an assembly graph (e.g., a *de Bruijn graph*^1^) are labeled with the number of times their corresponding string has been observed in the input reads.^2^^,^^3^^,^^4^^,^^5^ As another example, in RNA transcript assembly, many tools use *splice graphs* whose nodes (corresponding to exons) and arcs (corresponding to exon junctions) are labeled with their RNA-seq read abundances.^6^ Given these abundances, a solution to the assembly problem can be modeled as a *flow decomposition* into weighted paths or walks induced by such abundance values. In the case of perfect data, the superposition of these weighted walks matches the given flow. As a complication, a flow typically admits a heap of different flow decompositions. This ambiguity is a common issue in the assembly problem (see e.g.,^7^), and hence research has focused on reporting only so-called *safe walks* (modeling *contigs* output by modern assemblers), which are partial solutions that are common to all solutions, and hence must also be part of the true DNA or RNA sequence.^8^

**Figure 1:**
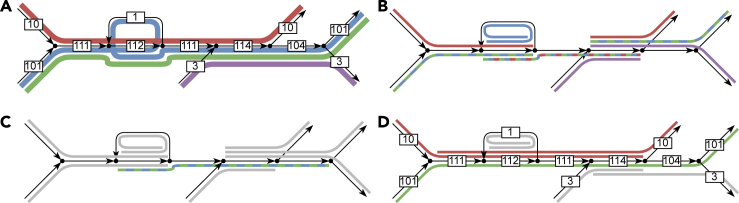
An assembly graph and its various -tigs All maximal structural contigs are subwalks of flowtigs. Flowtigs produce longer contigs especially for more abundant genomes, as they are not interrupted when meeting a low-abundant genome. (A) Excerpt of an example assembly graph built from four genomes with abundances 1 (in blue), 3 (in violet), 10 (in red), and 100 (in green). We assume that the genomes overlap nowhere else and that each genome is circular. (B) The maximally extended contigs (i.e., unitigs univocally extended forwards and backwards). The colors are chosen based on the genomes the contigs are subwalks of, whereas dashed contigs are subwalk of multiple genomes. These are arcs extended forwards as long as there is a unique outgoing arc, and backwards as long as there is a unique incoming arc. (C) The maximal structural contigs (i.e., safe walks w.r.t. arc-covering collections of closed walks). The walks that have not changed compared to maximally extended contigs are greyed out, and colors are chosen based on subwalks again. This contig is safe, because all arcs leaving it are connected only to its first node, and all arcs entering it are (reverse) connected only to its last node. (D) The maximal flowtigs (i.e., safe walks w.r.t. flow decompositions into weighted closed walks). The walks that have not changed are greyed out, and colors are chosen based on subwalks again. Note that the green genome can now be completely reconstructed through a single contig, and the red genome can be almost completely reconstructed. This is possible because they are the most abundant genomes.

For splice graphs in RNA transcript assembly (which are directed acyclic graphs, *DAGs*), Ma et al.^9^ gave the first algorithm to decide when a given set of arcs is *safe* for flow decompositions, i.e., when the arcs in the set appear in some and the same, path of any flow decomposition of the flow in the splice graph. When the arcs in the set form a path, Khan et al.^10^ improved the algorithm of Ma et al.^9^ from quadratic time to linear time, using a simple characterization of such *safe paths* via a notion of *excess flow* (the flow on the first arc minus the flow leaking from the internal nodes of the path). This also led to an optimal O(mn)-time algorithm identifying *all* maximal safe paths for flow decompositions in DAGs,^10^ where *n* and *m* denote the number of nodes and arcs in the graph, respectively. The experimental results on perfect splice graphs from Khan et al.^10^ show that safe paths for flow decompositions cover around 18% more of the ground-truth RNA transcripts than paths that are safe based only on the graph structure. Specifically, Khan et al. compare against *extended contigs*,^10^ i.e., unitigs extended forwards as long as the nodes have unit out-degree, and backwards as long as the nodes have unit in-degree, also known as *Y-to-V contigs*^7^^,^^11^^,^^12^ in the context of genome assembly, where they are close to optimal,^8^ see Figure 1B for an example. A reason behind the improvement is that the differences between the abundance levels of the different RNA transcripts in a sample can be multiple orders of magnitude.^10^^,^^13^ As such, abundant transcripts have a large *excess flow* and can span over branching nodes that would otherwise break a unitig or an extended contig.

Despite these theoretical and experimental results on safe paths for flow decompositions in DAGs, the analogous theory for non-acyclic assembly graphs is currently lacking. Motivated by the above results for acyclic splice graphs, it is natural to include also abundance of information to further restrict the set of assembly solutions in general graphs, to obtain longer safe walks, potentially leading to longer contigs in practice.

In this article, we choose metagenomic assembly as the application area to define a concrete notion of genome assembly solution as a flow decomposition and to evaluate the potential benefits of safe walks for flow decompositions compared to maximal safe walks relative only to the graph structure (*structural contigs*). Metagenomic assembly is crucial for understanding the functionality and composition of various microbiomes containing bacteria, archaea, viruses, and single-cell eukaryotes. These microorganism communities are ubiquitous and can be found in multiple ecosystems and multi-cellular organisms, including soil, seawater, the human digestive system, genitals, and others.^14^ While there are dedicated metagenomic assemblers for short-read sequencing data (e.g., metaSPAdes^4^ and Megahit^3^), there are still distinctive challenges compared to, e.g., single-genome assembly, that are still not completely resolved due to the greatly varying abundances of the species in the sample, and the intra- and inter-species repeats (see e.g.,^15^).

With respect to long-read sequencing techniques, the metagenomic assembly problem still raises some challenges, for example when dealing with low abundance levels of high strain variation species or bacterial species.^16^ Furthermore, the higher cost^17^ and larger variance in read length for PacBio HiFi technology^18^ as well as the large variance in coverage,^5^^,^^18^ presents additional issues. While partially alleviated by longer reads, intra-genomic and inter-genomic repeats and intra- and inter-species heterogeneity remain challenging.^5^^,^^16^

In addition to being an important problem, the metagenomic assembly problem also lends itself nicely to a flow-based theoretical formulation. As in the case of RNA transcripts, the species abundances in a typical metagenomic sample vary by multiple orders of magnitude,^19^^,^^20^^,^^21^^,^^22^ and this can similarly allow safe walks from more abundant genomes to safely continue over crossings with less abundant genomes. For example, if a branching node has two incoming arcs with abundances 10 and 100, and two outgoing arcs with abundances 90 and 20, then there it is certainly safe to continue from the abundance-100 arc to the abundance-90 arc. See Figure 1D for an example, and compare with Figures 1B and 1C where extended contigs and structural contigs are shorter than flowtigs.

Metagenomic assemblers usually employ overlap graphs^23^ or de Bruijn graphs,^1^ from which they compute unitigs^4^^,^^5^^,^^17^^,^^24^^,^^25^ as maximal safe paths and then extend them using possibly unsafe heuristics. Unitigs are non-branching paths in the assembly graph. They are *safe*, i.e., they are guaranteed to be subwalks of some genome in the metagenome under the assumption that all genomes are closed walks in the assembly graph such that each arc is covered by some genome, and that the assembly graph is error-free (*arc-centric model*). In this context, error-free setting means that the metagenome is sequenced completely and without errors and that the *k*-mer abundances reflect exactly how often each *k*-mer appears in the metagenome. Specifically, the *k*-mer abundance is the exact sum of occurrences in each copy of each genome in the metagenome. Recently, Acosta, Mäkinen and Tomescu^26^ have shown that there are longer walks that are safe based on the graph structure, w.r.t. solutions defined as arc-covering collections of closed walks. They have characterized a safe walk *W* by the conjunction of two conditions, and shown that these are both necessary and sufficient. First, there must be no cycle in the graph that contains a subwalk of *W*, but none of its prefixes and suffixes. Such cycles are also called *forbidden paths*.^8^ Second, there must be a node *v* in the graph such that all cycles through *v* have *W* as subwalk. Acosta, Mäkinen and Tomescu have shown that these maximal structure-based safe walks can be computed in $O\left(m^{2}+n^{3}\;\log\;n\right)$ time in node-centric assembly graphs and in $O\left(m^{2}n\;\log\;n\right)$ time in arc-centric assembly graphs. Later, Cairo et al.^27^ have improved this bound for node-centric assembly graphs to O(mn+o), where *o* is the total length of the maximal safe walks.

While these articles close the question of what are the longest walks that can safely be reported from an assembly graph by considering only its structure (assuming perfect coverage), when introducing abundances the field is much less explored. In the case of a flow decomposition into a single walk where all arcs have abundance 1, i.e., the decomposition into an *Eulerian walk*, the safe walks have been characterized.^28^ This model however is not realistic, as the abundance restricted to be only 1 forbid repeats, which are a common structure in genomes. Additionally, a model with restricting the flow decomposition to a single closed walk (but without restricting the abundances to value 1) was considered before,^29^ but not under the perspective of safety. Such a model however would not be useful for metagenomic assembly, as it assumes just one genome.

In line with previous studies assessing the potential benefits of using longer safe paths or walks,^8^^,^^10^^,^^30^ in this article, we assume an error-free setting. As such, each circular genome (with its abundance) corresponds to a weighted closed walk in the graph, and thus the superposition of these walks induces a flow where the *flow conservation property* holds at every node, i.e., the sum of incoming flow equals the sum of outgoing flow. Given only the graph and its flow, since we have no further information to decide which flow decomposition is the correct one, a metagenomic assembly is then *any* decomposition of the flow (into weighted closed walks). We study the problem of finding the safe walks in this model, i.e., finding those walks *W* such that for any flow decomposition $D=\left\{D_{1},\ldots ,D_{k}\right\}$ into closed weighted walks, there exists a closed walk $D_{i}$ such that *W* is a subwalk of $D_{i}$.

## Results

In this work, we make several theoretical and practical contributions.

### Characterization of safe walks

We provide the first complete characterization of safe walks for flow decompositions into weighted closed walks in general graphs via *flowtigs*. Such complete characterization was formerly only known for flow decompositions into weighted source-to-sink paths in DAGs.^10^ More specifically, we prove that a walk *W* is safe for an instance (G,f) of the flow decomposition problem if and only if it is a flowtig, where *G* is a strongly connected directed graph and *f* is a flow function mapping edges to positive rational numbers. Surprisingly, the same characterization as for DAGs (suitably generalized) still works in general graphs, and it is simpler than the one of the structural contigs.^26^^,^^27^ Indeed, our characterization of flowtigs involves only local and simple properties (e.g., a particular set of arcs that interact with the walk in question and their flow values), as opposed to structural contigs, which are characterized by forbidden paths, a more global property related to the existence of a particular type of path between internal nodes of the walk. This makes flowtigs verifiable in linear time in their length and simpler to integrate into real assemblers.

We remark that even though the characterizations are the same when moving from DAGs to general graphs, the proof of correctness becomes significantly more complicated. Khan et al.^10^ proves the unsafety of a non-flowtig *W* by constructing an arbitrary avoiding flow decomposition by taking any leaving arc of *W*. In general graphs, no leaving arc can be taken, because a wrong decision early on might force us to traverse *W* in the future, even though it would have been possible to avoid it otherwise. We show how to overcome this by using the leaving arcs of *W* in a particular way, thus allowing us to construct such avoiding decompositions.

### Enumeration algorithms

Further, assuming perfect data, we introduce an algorithm that can identify all maximal flowtigs (possibly including duplicates) in O(mn) time in the worst case, inspired by that of Khan et al..^10^ It first computes a flow decomposition of total size O(mn) and then identifies the subwalks of the decomposition that are maximal flowtigs. The algorithm then reports the flow decomposition and a list of start and endpoints of maximal flowtigs within the decomposition. However, the actual time complexity of the algorithm is linear in the size of the flow decomposition, which is often much smaller than quadratic in practice, making it competitive (in terms of runtime) against e.g., computing unitigs or extended contigs. We also give a family of graphs that contain Θ(mn) distinct maximal flowtigs, proving that our algorithm is optimal for worst case instances. More formally, given a flow graph (G,f) having *n* vertices and *m* arcs, all its maximal flowtigs can be identified in O(||D||)⊆O(mn) time and space, where D is some flow decomposition.

For real data, we adapt the definition of flowtig to a more realistic setting, showing that flowtigs can be used as a heuristic to find longer safe walks in assembly graphs built from real datasets. Note that in these graphs flow conservation does not necessarily hold in every node, making them more challenging to work with from a theoretical perspective. We develop an algorithm running in O(m·out) time, where out denotes the length of all the flowtigs, including non-maximal ones. We show that $out=O\left(m^{1+mU/u}\right)$, where *U* denotes the largest flow value and *u* the smallest flow value in the arcs of *G*. Further, we show that although this runtime is exponential, in practice the algorithm is fast even on large graphs. This algorithm resembles the one presented in^8^ enumerating all maximal omnitigs of a genome graph. On the whole, given an assembly graph built from real data, it is possible to find all its flowtigs in $O\left(m^{2+mU/u}\right)$ time. Our algorithm considers an arc of the graph and, in a depth-first fashion, tries all possible arc extensions that preserve safety. When there are no more safe extensions, it stores the current flowtig and continues the search in another branch. When the search on this arc finishes, it performs the same search on another arc. We also describe a heuristic to filter some flowtigs from the output in order to improve the accuracy of the reported safe walks while essentially maintaining the same metrics of assembly contiguity.

### Application to metagenomic assembly

On the practical side, we conduct experiments on real data, comparing flowtigs against unitigs, extended contigs, and structural contigs, on various metagenomic datasets (see Table 1). We first focus on the effects introduced by using flowtigs, and in line with previous studies for the DAG case,^9^^,^^10^ we run our experiments on error-free data. This shows that flowtigs provide consistently better assembly contiguity than unitigs on all tested datasets (see Table 2). Moreover, the contiguity improvement that flowtigs bring over unitigs is 4–14 × larger than what extended unitigs or structural contigs provide over unitigs.

**Table 1:** Features of the datasets

	simple7	medium20	complex32	JGI	HMP	Zymo
#genomes	7	20	32	26	23	17
#genera	6	19	26	22	19	17
#bases	20.8M	72.8M	120M	105M	83.0M	93.2M
#DBG nodes	10.8k	42.4k	459k	169k	71.6k	3,820k
#DBG arcs	16.9k	65.8k	695k	260k	111k	4,930k
flow decomposition size	301k	614k	9,550k	2,650k	999k	–

The DBG is an arc-centric de Bruijn graph of order k=31. The flow decomposition is that computed by our enumeration algorithm (the Zymo assembly graph does not admit flow decompositions as the data is not perfect).

**Table 2:** Assembly contiguity and computation time of various -tigs on simulated data

dataset	metric	unitigs	extended contigs	structural contigs	flowtigs
simple7	EA50	21.8k	21.9k (0.105%)	21.9k (0.105%)	24.7k (13.0%)
	EA75	10.5k	10.8k (2.38%)	10.8k (2.38%)	12.2k (15.6%)
	gen. frac.	99.75%	99.99%	99.99%	99.99%
	time (s)	85.5	3.08	7.52	5.32
medium20	EA50	22.5k	22.8k (1.11%)	22.8k (1.11%)	25.6k (13.5%)
	EA75	10.3k	10.7k (3.24%)	10.7k (3.24%)	12.3k (18.9%)
	gen. frac.	99.55%	99.97%	99.97%	99.98%
	time (s)	200	11.0	81.0	17.2
complex32	EA50	11.1k	11.3k (1.87%)	11.3k (1.87%)	12.5k (13.0%)
	EA75	535	662 (23.7%)	662 (23.7%)	1.07k (100.4%)
	gen. frac.	94.24%	99.85%	99.85%	99.77%
	time (s)	267	20.4	28,700	164
JGI	EA50	16.5k	16.8k (2.17%)	16.8k (2.17%)	19.3k (17.0%)
	EA75	4.67k	4.88k (4.56%)	4.88k (4.56%)	5.89k (26.2%)
	gen. frac.	99.14%	99.96%	99.96%	99.97%
	time (s)	348	17.1	5,740	41.8
HMP	EA50	19.4k	19.6k (0.952%)	19.6k (0.952%)	22.1k (13.9%)
	EA75	8.62k	8.79k (2.03%)	8.79k (2.03%)	9.86k (14.4%)
	gen. frac.	99.22%	99.91%	99.91%	99.95%
	time (s)	211	12.2	216	20.2

The EA50 and EA75 metrics are an extension of the NGA50 and NGA75 metrics that are robust against overlapping and repeated contigs; in parentheses, we give the improvement of the respective contigs over unitigs. “gen. frac.” is the percentage of the metagenomic reference that is covered by the contigs. Since we work with perfect data, this should always be exactly 100%. But since QUAST uses approximate alignment, it is not. See details about implementation and evaluation in the STAR Methods section. “k” is the SI multiplier by 1,000. Unitigs are computed with BCALM2.^31^ All other algorithms take unitigs as input, but for readability we do not include the computation of unitigs in their performance metrics. The computation of flowtigs contains also the run of a separate tool that transforms the node-centric DBG output by BCALM2 into an arc-centric one.

The algorithm we propose assuming error-free data are very fast, taking only 2 min and using less than 4 GiB of memory to execute on the largest (compacted) graph with 459 thousand nodes and 695 thousand arcs (complex32 dataset). See Table 3.

**Table 3:** Performance for computing various -tigs on simulated data (memory and time)

dataset	metric	unitigs	extended contigs	structural contigs	flowtigs
simple7	time (s)	85.5	3.08	7.52	5.32
	RAM (MiB)	1,319	14.7	13.6	118
medium20	time (s)	200	11.0	81.0	17.2
	RAM (MiB)	3420	40.6	42.3	349
complex32	time (s)	267	20.4	28,700	164
	RAM (MiB)	5,240	219	226	3,920
JGI	time (s)	348	17.1	5,740	41.8
	RAM (MiB)	5,520	102	104	993
HMP	time (s)	211	12.2	216	20.2
	RAM (MiB)	4,250	58.8	84.1	527

Values are rounded to the three most significant digits. Unitigs are computed by BCALM2^31^ which uses external memory and runs in parallel. All other algorithms take unitigs as input, but for readability we do not include the computation of unitigs in their performance metrics. The computation of flowtigs contains also the run of a separate tool that transforms the node-centric DBG output by BCALM2 into an arc-centric one, since the flowtigs tool cannot directly read the output of BCALM2.

Second, to evaluate the potential of flowtigs on real data, we conducted experiments on the ZymoBIOMICS Gut Microbiome Standard dataset (see Table 4). Using the practical enumeration algorithm we show that flowtigs also provide consistently better assembly contiguity than unitigs in this case. Specifically, our improvement in assembly contiguity over unitigs is up to one order of magnitude larger than the improvement of extended contigs over unitigs. We achieve this while having around less than half of the misassemblies contained by unitigs. Our algorithm for real data is not as fast as the one for simulated data is compared to, e.g., extended unitigs. Nevertheless, it runs in less than 6 min and requires less than 10 GiB of memory to execute in the complex32 dataset (see Table 5).

**Table 4:** Assembly contiguity on real data

k	metric	unitigs	extended contigs	0-flowtigs	7-flowtigs	(0,7)-flowtigs	(0,10)-flowtigs
31	EA10max	778	1,440 (85.5%)	8,200 (954%)	7,900 (916%)	5,640 (625%)	3,820 (391%)
	EA25max	149	283 (89.9%)	3,250 (2,080%)	3,190 (2,040%)	2,380 (1,500%)	1,030 (593%)
	EA40max	68	150 (121%)	779 (1,050%)	750 (1,000%)	540 (694%)	308 (353%)
	EA50max	0	118	383	364	232	0
	genome fraction (%)	41.2	65.6	60.1	59.6	54.9	47.6
	misassemblies	287	427	1,290	755	98	61
51	EA10max	864	1,680 (94.6%)	10,200 (1,080%)	9,900 (1,050%)	6,750 (681%)	4,400 (409%)
	EA25max	182	356 (95.6%)	3,930 (2,060%)	3,860 (2,020%)	2,840 (1,460%)	1,150 (530%)
	EA40max	101	209 (107%)	937 (827%)	910 (801%)	674 (567%)	392 (288%)
	EA50max	98	173 (76.5%)	495 (405%)	477 (387%)	320 (227%)	0
	genome fraction (%)	66.4	66.3	59.8	59.5	54.8	47.1
	misassemblies	286	449	1,530	1,080	176	98
101	EA10max	1,070	2,230 (110%)	13,900 (1,200%)	13,300 (1,150%)	9,560 (798%)	6,000 (463%)
	EA25max	288	567 (96.9%)	5,370 (1,760%)	5,290 (1,740%)	4,050 (1,310%)	1,670 (479%)
	EA40max	199	368 (84.9%)	1,360 (583%)	1,340 (572%)	1,050 (431%)	678 (241%)
	EA50max	198	312 (57.6%)	803 (306%)	782 (295%)	587 (196%)	0
	genome fraction (%)	65.8	65.6	59.4	59.2	55.1	47.3
	misassemblies	417	619	2,060	1,630	254	123
251	EA10max	1,880	4,150 (121%)	23,900 (1,170%)	22,900 (1,120%)	17,200 (814%)	11,600 (518%)
	EA25max	649	1,330 (105%)	9,650 (1,390%)	9,550 (1,370%)	7,660 (1,080%)	4,340 (568%)
	EA40max	498	873 (75.3%)	2,910 (484%)	2,840 (470%)	2,350 (372%)	1,700 (240%)
	EA50max	497	738 (48.5%)	1,840 (270%)	1,790 (260%)	1,430 (187%)	0
	genome fraction (%)	63.7	63.6	59.0	58.9	55.4	48.7
	misassemblies	571	641	1,780	1,400	240	149
501	EA10max	3,690	7,990 (119%)	40,100 (985%)	39,200 (962%)	29,800 (706%)	20,400 (452%)
	EA25max	1,330	2,830 (113%)	16,600 (1,150%)	16,500 (1,140%)	13,600 (924%)	9,080 (583%)
	EA40max	998	1,770 (77.5%)	5,820 (484%)	5,680 (469%)	4,590 (360%)	3,370 (237%)
	EA50max	996	1,470 (47.6%)	3,630 (264%)	3,520 (253%)	2,820 (183%)	0
	genome fraction (%)	61.9	61.8	58.8	58.7	55.5	49.1
	misassemblies	591	535	1,650	1,420	235	137
1001	EA10max	8,290	18,400 (122%)	72,400 (773%)	69,500 (739%)	51,400 (520%)	36,700 (342%)
	EA25max	2,960	6,680 (125%)	30,900 (944%)	30,300 (924%)	24,200 (716%)	18,100 (509%)
	EA40max	2,000	3,700 (84.9%)	13,200 (561%)	12,600 (528%)	8,630 (332%)	6,100 (205%)
	EA50max	2,000	3,000 (50.2%)	6,810 (241%)	6,530 (227%)	5,110 (156%)	0
	genome fraction (%)	60.3	60.2	58.7	58.6	54.50	48.7
	misassemblies	409	357	1,560	1,250	287	163

Similarly to Table 2, we include EA10, EA25, EA40, and EA50 metrics, where in parentheses we give the improvement of the respective contigs over unitigs; we also include the genome fraction, which denotes the percentage of the metagenomic reference that is covered by the contigs; details concerning the computation of unitigs as well as the technicalities about the construction of the DBG can be found in the description of Table 2. Since we are working with real data, we include the number of “misassemblies” for every type of contig that QUAST reports. Here, we do not include unitigs in the output - the idea of the filter is to remove flowtigs containing misassemblies while maintaining good EA metrics, and thus it would defeat the purpose of the filter to add unitigs to the output since they contain misassemblies and do not improve any EA.

**Table 5:** Time and memory resources of the real data algorithm

k	metric	unitigs	ext. contigs	0-flowtigs	7-flowtigs	(0,7)-flowtigs	(0,10)-flowtigs
31	time (s)	1,010	44.0	309	400	323	189
	RAM (MiB)	2.77k	1.32k	10.7k	10.3k	10.2k	10.1k
51	time (s)	1,040	57.6	364	267	246	167
	RAM (MiB)	3.82k	1.16k	9.70k	9.28k	9.23k	9.10k
101	time (s)	1,670	59.6	275	293	245	119
	RAM (MiB)	3.59k	916	7.65k	7.27k	7.20k	7.09k
251	time (s)	1,670	69.6	190	170	148	92.3
	RAM (MiB)	3.62k	547	5.44k	4.92k	4.05k	3.98k
501	time (s)	2,130	69.8	133	123	98.6	70.0
	RAM (MiB)	3.75k	339	4.56k	4.08k	2.33k	2.27k
1001	time (s)	1,800	65.4	97.9	94.0	89.4	57.9
	RAM (MiB)	4.24k	202	3.64k	3.22k	1.54k	1.53k

The impact of different k-mer sizes in time and space requirements of Algorithm 1.

We thus present evidence that flowtigs (i.e., paths/walks with the positive excess flow) can be used as a theory-rooted heuristic, both as a standalone technique to compute contigs and to complement current path-finding techniques used by state-of-the-art assemblers. While such applications are outside of the scope of this article, the two main favorable properties are: (i) Flowtigs are defined by a very simple property, which is also *local* to the nodes and arcs of the flowtig (i.e., excess flow). On the other hand, structural contigs rely on the global properties of the graph (the absence of forbidden paths), and thus a single false positive or false negative node or arc can have global effects. (ii) Flowtigs can be less sensitive to local errors than unitigs, extended and structural contigs because they take abundances into account. In this way, arcs with low abundance and hence on the verge of invalid deletion by an error-correction algorithm can be left untouched for flowtigs and will affect the resulting contigs only lightly because of their small abundance.

## Discussion

In this article, we introduce flowtigs and compare them to previous safe contigs. On all the analyzed datasets, flowtigs are the only safe contigs that exhibit non-negligible improvements over unitigs. For the first time in general graphs, we hope to have a notion of safe walks enjoying the same and in parts even better favorable robustness properties as unitigs and extended contigs, while potentially resulting in a much larger improvement in assembly contiguity.

In the future, we hope that we can obtain even longer safe walks than flowtigs by making use of more information that is usually available during a modern assembly process. Flowtigs use all information that is available through the structure of the assembly graph and the abundance values on the arcs. Hence, based just on this information, no further improvement can be obtained. However, in a modern assembly process, there is typically more information available that we have so far ignored. For example, in the most recent assembly of the human genome,^32^ an assembly graph was constructed using very accurate long reads (PacBio HiFi), and then ULTRA long-reads (Oxford Nanopore) were aligned to that graph. These longer reads were used to obtain very long contigs by heuristically bridging through even complex tangles in the assembly graph. Having proven their potential, it would be very interesting to see what information can safely be extracted from them in a theoretically sound model, e.g., by using them as subpath constraints for a flow decomposition.^33^ This could also result in an assembly pipeline that completely automates genome assembly such as that of Rautiainen et al.,^34^ but by using only safe algorithms. Such a pipeline would have the desirable property that it produces no errors given error-free data.

### Limitations of the study

#### Subwalks and contigs in metagenomics

The model of safety we propose for metagenomics is slightly different than previous models of safety (e.g.,^8^^,^^10^^,^^35^). In those models, when a walk of an assembly graph has the property of being safe, it can be inferred that the string spelled by that walk is somewhere present in the sample. With our definition of a subwalk, a walk being safe has a different interpretation. If the walk is a path then the string spelled by the path is present in a genome of the ground truth. If the walk is a recurring cycle W=C…CP where *C* is repeated a given number of times and *P* is a (possibly empty) path over the cycle starting in the same arc as *C*, then there is a collection of genomes in the sample with respective abundances containing subsequences that “sum up” to *W*, so it does not give information about a particular genome but of (possibly many) feasible different arrangements of a set of genomes.

**Figure 2:**
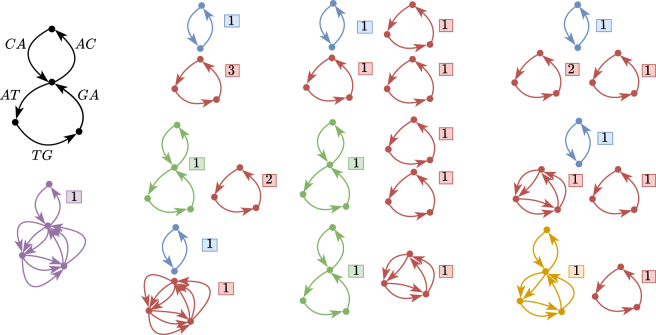
A simple assembly graph and its integral flow decompositions An arc-centric de Bruijn graph is shown on the top left; the three arcs of the bottom cycle all have weight three and those of the top cycle have weight one. The remaining figures depict all the flow decompositions of this graph; each closed walk has an associated weight depicted in a squared box and arcs drawn multiple times between the same pair of nodes mean that there is a closed walk in the flow decomposition that repeats that arc the number of times it is drawn. One reconstruction of the genomes is, for example, the blue genome CA with abundance one and three times the red genome ATG each with abundance.

In other words, it means that the set of genomes in the sample whose sequences support the arcs present in *C* is of the form $\left\{\left(D_{1},w_{1}\right),\ldots ,\left(D_{l},w_{l}\right)\right\}$, where l≥1 and every $D_{i}$ but one contains the genomic subsequence *C*, possibly repeated according to the flow constraints on the arcs of the graph, and where the other genome contains C…CP as subsequence where *C* can appear 0 or more times. As an example consider Figure 2. We see that the string s=ATGATGATG spelled by the bottom recurring cycle is safe and that it is a substring of all the flow decompositions of the graph (according to our definition of substring given in the main matter). According to the more conventional definition of substring over circular strings, i.e., a string *X* is a substring of *W* if *X* is a substring of some rotation of *W*, then our excess flow characterization fails; for example, the flow decomposition that decomposes the bottom cycle with one cycle of weight two and one cycle of weight one does not contain *s* as a substring anywhere. However, using our definition of substring over closed walks, we can “fit” the sequence *s* in some closed walk of any flow decomposition.

Note that, even though our model here is not “safe” in the classic sense, it still heuristically fits better to real data. In real data, genomes are long and it is unlikely that any given cycle in the graph is a complete genome. Much more likely is that most of these cycles are repeats in a genome, and hence are handled correctly by our flowtigs. Therefore, we deem our formulation as a more practical one than the classic definition of safety.

## Resource availability

### Lead contact

Requests for further information and resources should be directed to and will be fulfilled by the lead contact, Alexandru Tomescu (alexandru.tomescu@helsinki.fi).

### Materials availability

This study did not generate new materials.

### Data and code availability

• This article analyzes existing, publicly available data. The required accession numbers for the datasets are listed in the key resources table. The datasets are also available on Zenodo.^36^
• Our source code^37^ is available on GitHub (https://github.com/algbio/flowtigs) and on Software Heritage^38^ under the BSD-2-Clause license. The repository with the code to reproduce all the experiments is available on GitHub (https://github.com/elieling/safe-paths-with-flowtigs).

## Acknowledgments

This work was partially funded by the 10.13039/501100000781European Research Council under the European Union’s Horizon 2020 research and innovation programme (grant agreement No. 851093, SAFEBIO), and partially by the 10.13039/501100002341Research Council of Finland (grants No. 322595, 352821, 346968, 358744). Additionally, Shahbaz Khan is funded by the Startup Research Grant SRG/2022/000801 by DST SERB, Government of India. We thank the Finnish Computing Competence Infrastructure (FCCI) for supporting this project with computational and data storage resources.

## Author contributions

All authors conceived the project, developed the methods, interpreted the results, and wrote the article. E.I and S.S. wrote the software.

## Declaration of interests

The authors declare no competing interests.

## STAR★Methods

### Key resources table

**Table:** 

REAGENT or RESOURCE	SOURCE	IDENTIFIER
**Biological samples**		

Simple7, Medium20, Complex32	Shafranskaya et al.^22^	https://doi.org/10.3389/fmicb.2022.981458
JGI	Singer et al.^20^	https://doi.org/10.1038/sdata.2016.81
HMP	Liu et al.^21^	https://doi.org/10.1186/s40168-020-00937-3
Zymo D6331 PacBio HiFi reads	Zymo Research https://zymoresearch.eu/products/zymobiomics-gut-microbiome-standard	SRA accession SRR13128014

**Software and algorithms**		

Flowtigs code	This paper	https://github.com/algbio/flowtigs

**Other**		

Simulated abundances for JGI and HMP	This paper	https://doi.org/10.5281/zenodo.13839057

### Method details

#### Preliminaries

A *directed* graph *G* is a tuple (V,E), where *V* is the set of *nodes* and *E* the set of *arcs*. We allow graphs to have parallel arcs, that is, two vertices may be connected by more than one arc, but we forbid self-loops (since they can be replaced by a path of length two). As such, for an arc e∈E from *u* to *v*, we define t(e):=u to be its *tail*, and h(e):=v to be its *head*. The *in-neighbourhood* of a node v∈V is the set $N^{-}\left(v\right)$ of its incoming arcs, and the *out-neighbourhood* is the set $N^{+}\left(v\right)$ of its outgoing arcs.

A *walk W* is a sequence of nodes alternated with arcs $W:=\left(v_{1},e_{1},\ldots ,v_{l-1},e_{l-1},v_{l}\right)$ such that $t\left(e_{i}\right)=v_{i}$ and $h\left(e_{i}\right)=v_{i+1}$. We denote by ${\#}_{W}\left(e\right)$ the *multiplicity* of *e* in *W* (i.e., the number, possibly zero, of occurrences of *e* in *W*). A *u-v walk* is a walk such that $t\left(e_{1}\right)=u$ and $h\left(e_{l}\right)=v$. A *closed walk* is a *v*-*v* walk for some v∈V. A *path* is a walk where all $v_{i}$ are unique. For paths, $v_{l}=v_{1}$ is allowed, in which case it is a *closed path* (we interchangeably use the term *cycle*). In what follows, we will be usually working with walks along cycles, so we define a *recurring cycle* with respect to a cycle *C* to be a walk beginning with *C* followed by a (possibly empty) prefix of any number of concatenations of *C* with itself. For a walk *W*, we denote by |W| its number of arcs. For a set of walks $W=\left\{W_{1},\ldots ,W_{k}\right\}$ we denote its *total size* (number of arcs) by $\left|\left|W\right|\right|=\left|W_{1}\left|+\cdots +\right|W_{k}\right|$.

If $W_{1}=\left(u_{1},e_{1},\ldots ,e_{l-1},u_{l}\right)$ and $W_{2}=\left(v_{1},e_{1}^{\prime },\ldots ,e_{p-1}^{\prime },v_{p}\right)$ are walks such that $u_{l}=v_{1}$, then $W_{1}W_{2}:=\left(u_{1},e_{1},\ldots ,e_{l-1},u_{l},e_{1}^{\prime },\ldots ,e_{p-1}^{\prime },v_{p}\right)$ denotes their concatenation. If $e=\left(u_{l},x\right)$, we define the concatenation of a walk with an arc as $W_{1}e:=\left(u_{1},e_{1},\ldots ,u_{l-1},e_{l-1},u_{l},e,x\right)$. We say that for e∈W the *e*-prefix of *W* is the prefix of *W* until the last occurrence of *e* in *W*. A graph is *strongly connected* if for every pair of nodes u,v∈V there is a *u*-*v* path.

A *flow f* in a graph G=(V,E) is a function $f:E\to Q^{+}$ such that for any node u∈V, its *incoming flow* $f_{in}\left(u\right):=\sum_{e\in N^{-}\left(v\right)}f\left(e\right)$ is equal to its *outgoing flow* $f_{out}\left(u\right):=\sum_{e\in N^{+}\left(v\right)}f\left(e\right)$ (*flow conservation*). Note that we assume flow conservation in *every* node (such flows are also called *circulations*, see e.g.,^39^^,^^40^).

A *decomposition* D of a *flow graph* (G,f) is a multiset of *weighted closed walks* $\left(D_{i},w_{i}\right),i\in \left\{1,\ldots ,\left|D\right|\right\}$ with an associated positive rational weight $w_{i}\in Q^{+}$, such that their superposition matches the flow *f*, i.e. $\forall e\in E:f\left(e\right)=\sum_{i}{\#}_{D_{i}}\left(e\right)\cdot w_{i}$. The *addition* $f=f_{1}+f_{2}$ of two flows is defined as $\forall e\in E:f\left(e\right)=f_{1}\left(e\right)+f_{2}\left(e\right)$ and the *subtraction* $f=f_{1}-f_{2}$ is defined as $\forall e\in E:f\left(e\right)=f_{1}\left(e\right)-f_{2}\left(e\right)$. The *multiplication* $f=k\cdot f_{1}$ of a flow with a scalar $k\in Q^{+}$ is defined as $\forall e\in E:f\left(e\right)=k\cdot f_{1}\left(e\right)$. The *induced flow* of a walk *W* is defined as $f\left(e\right):={\#}_{W}\left(e\right)$.

The following facts are well known and we refer the reader to e.g. the monographs^40^^,^^41^ for further details.

##### Lemma 1

If f and $f^{\prime }$ are flows, then $f+f^{\prime }$ is a flow and $f-f^{\prime }$ is a flow if it contains only positive values. A flow graph (G,f) has zero or more components all of which are strongly connected.

In order to define our safe walks, we first define subwalks of decomposing walks. For a walk *W* that is not closed we define a *subwalk X* to be a walk whose arcs are a substring of the arcs of *W*. For a closed walk *W* we define a *subwalk X* to be a walk whose arcs are a substring of any number of concatenations of *W* with itself. Specifically, *X* may be a substring of only *W*, or *X* starts with a (possibly empty) suffix of *W*, followed by zero or more repetitions of *W*, and ends with a (possibly empty) prefix of *W*. Note that this definition of subwalks of closed walks differs from the usual definition that does not allow *X* to repeat *W*. We are interested in all the *maximal* safe walks, that is, safe walks such that by extending them with a single arc (in the beginning or the end) renders the walk unsafe.

##### Definition 2 (Safety)

Let (G,f) be a flow graph. Then a walk W is safe in (G,f), if for each decomposition D into weighted closed walks of (G,f), it holds that there is some closed walk D∈D, such that W is a subwalk of D. A maximal safe walk is a safe walk that can not be extended without losing the safe property.

We assume onwards that every connected component of *G* is always different from a single cycle, since in those cases the assembly problem becomes trivial. Further, this case does not introduce extra complexity to our safety framework but just a simple corner case: we let the walk defined by such a cycle to be maximally safe.

#### Characterising safe walks

We begin by recalling some key notions introduced by Khan et al.^10^ for the DAG case. First, we define an arc to be *leaving* from a walk if it is out-going from one of its internal nodes.

##### Definition 3 (Leaving arc^10^)

A leaving arc of a walk $W=\left(v_{1},e_{1},\ldots ,v_{l-1},e_{l-1},v_{l}\right)$ is an arc e such that $\exists i\in \left\{2,\ldots ,l-1\right\}:t\left(e\right)=t\left(e_{i}\right)$ and $h\left(e\right)\neq h\left(e_{i}\right)$.

Next, we recall the notions of *leakage* of a walk (as the total amount of flow that leaves the walk before it ends), and the notion of *excess flow* of a walk (as the flow entering the walk through its first arc, minus its leakage). These concepts will be crucial for showing that a given walk is safe or unsafe. Note that our definition below applies to general graphs, not only to DAGs as in.^10^ For example, if there is a leaving arc from a node *v* for a walk *W*, then its flow value contributes to the leakage of *W* as many times as the number of occurrence of *v* as internal node of *W*.

##### Definition 4 (Leakage and Excess flow^10^)

*The leakage*leakage(W)*and the excess flow*excess(W)*of a walk*$W=\left(v_{1},e_{1},\ldots ,v_{l-1},e_{l-1},v_{l}\right)$*are defined as:*$\text{leakage}\left(W\right):=\sum_{i=2}^{l-1}f_{out}\left(v_{i}\right)-f\left(e_{i}\right),\text{excess}\left(W\right):=f\left(e_{1}\right)-\text{leakage}\left(W\right)$.

The core idea of the characterisation of safe walks for weighted flow decompositions into source-to-sink paths in DAGs by Khan et al.^10^ is to ask where the incoming flow of a given walk can go: certainly, it can flow through the entire walk, but it can also flow through its leaving arcs. The relation between the incoming flow and the leakage is what fully characterises safe walks in DAGs, in fact, safe paths.

For general flow graphs, we define *flowtigs* as those walks with positive excess flow; see Figure 3 for examples.

**Figure:**
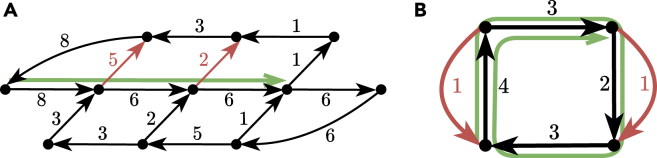
Examples of flowtigs The walk *W* is represented in green and its leaving arcs in red. The numbers denote the flow values of the arcs. (A) *W* is a path with leakage(W)=5+2 and excess(W)=8−7=1. (B) *W* is a recurring cycle with leakage(W)=1+1+1 and excess(W)=4−3=1.

##### Definition 5 (Flowtig)

A walk W is a flowtig if excess(W)>0.

Our first result below allows us to focus on only two particular types of walks when reasoning about safety, as it imposes restrictions on the shape of any potential safe walk.

##### Lemma 6

Any cycle followed or preceded by a single arc

1 is not safe, and
2 its excess flow is non-positive.

Proof.

Let W=PC be a walk consisting of a path made up from a single arc P=(u,e,v) followed by a cycle $C=\left(v,e_{1},\ldots ,v\right)$. The case of a cycle followed by an arc is completely symmetric.

For 1), let $w=\min_{e\in C}f\left(e\right)$ and let D=(C,w) be a closed walk (in fact, a cycle) of weight *w*. The walk W=PC is not a subwalk of any closed walk belonging to a decomposition containing *D*, thus *W* is unsafe.

For 2), we first observe that $f\left(e\right)\leq f_{in}\left(v\right)$ holds (in general, equality does not hold because other arcs may be entering *v*). Due to flow conservation, $f_{in}\left(v\right)$ units of flow must be carried from *v* along *C*, and they must eventually exit from *C* (at latest, in *v*). Note that the nodes of *C* are exactly the internal nodes of the walk *W*. Thus $f_{in}\left(v\right)\leq \text{leakage}\left(W\right)$ (in general equality does not hold because other arcs may be entering in nodes of *C*). Thus $f\left(e\right)\leq f_{in}\left(v\right)\leq \text{leakage}\left(W\right)$, and thus *W* has non-positive excess flow.

Consequently, any extension of walks of this form are unsafe, and, therefore, any safe walk is either a recurring cycle or a path (see Figure 3). Importantly, such walks cannot contain leaving arcs of themselves, a fact that we will use in the next results.

##### Corollary 7

Any safe walk for an instance (G,f) of the flow decomposition problem into closed walks is either a recurring cycle or a path.

To prove our characterisation we will routinely build closed walks that contain a proper prefix of the given walk and then escape via one of its leaving arcs. The following definition addresses this need.

##### Definition 8 (Escaping walk)

An escaping walk of a walk $W=\left(v_{1},e_{1},\ldots ,v_{l-1},e_{l-1},v_{l}\right)$ is any closed walk containing a non-empty prefix of W, but not containing W as a subwalk.

We can now show that whenever a walk has positive leakage and does not contain any of its leaving arcs, it admits an escaping walk.

##### Lemma 9

Let (G,f) be a flow graph and let $W=\left(v_{1},e_{1},\ldots ,v_{l-1},e_{l-1},v_{l}\right)$ be a walk with leakage(W)>0 such that W does not contain any leaving arc of W itself. Then, for any leaving arc l of W and for any occurrence of t(l) in W, there is a corresponding escaping walk $W_{l}$ traversing W until that occurrence of t(l).

Proof.

Let *l* be any leaving arc of *W* (which must exist since leakage(W)>0). Let $W_{1}$ be the walk along *W* from $v_{1}$ to any occurrence of t(l) in *W* and let $P_{2}$ be a path from h(l) to $v_{1}$. Such path $P_{2}$ always exists by Lemma 1 (note that t(l) and $v_{1}$ are in the same component). In case $P_{2}$ uses any additional leaving arcs of *W*, we let *l* to be the last leaving arc occurring in $P_{2}$, and change $P_{2}$ to be the path from h(l) to $v_{1}$. Moreover, for any occurrence of t(l) in *W*, we can change the prefix $W_{1}$ to be until that occurrence of t(l).

Now, for any $W_{1}$ we can define the closed walk $W_{l}:=W_{1}lP_{2}$. Note that $W_{l}$ does not contain *W* as subwalk because $W_{1}$ is a proper prefix of *W* and the only occurrence of $v_{1}$ in $P_{2}$ is at the end of $P_{2}$, therefore $W_{l}$ is an escaping walk of *W*. ∎

We now have everything needed to show our theorem characterising safe walks. To show that a walk *W* with non-positive excess flow is unsafe, we give a constructive argument that builds a flow decomposition where *W* is not a subwalk of any closed walk of the decomposition. We handle separately the two cases from Corollary 7 for a walk to be safe. The idea is to use Lemma 9 which will allow us to escape from *W* in a way that we can control both the decrease in the flow value of the first arc of *W* and in the leakage of *W*.

##### Theorem 10 (Safety via flowtigs)

A walk W is safe for an instance (G,f) of the flow decomposition problem if and only if it is a flowtig.

Proof.

Let $W:=\left(v_{1},e_{1},\ldots ,v_{l-1},e_{l-1},v_{l}\right)$. We prove both directions of the statement.

(⇐) Suppose *W* is a flowtig, i.e., $f\left(e_{1}\right)>\text{leakage}\left(W\right)$. We prove that *W* is also safe for (G,f). Each decomposition of (G,f) can carry at most leakage(W) flow through closed walks that contain $e_{1}$ but do not have *W* as subwalk. Therefore, since $f\left(e_{1}\right)>\text{leakage}\left(W\right)$, it follows that each decomposition must contain a closed walk having *W* as a subwalk, so *W* is safe for (G,f).

(⇒) Suppose *W* is safe, and assume for contradiction that *W* is not a flowtig, i.e. $f\left(e_{1}\right)\leq \text{leakage}\left(W\right)$. We argue separately over the two possible cases shown in Corollary 7 for a walk to be safe. In both cases we make use of Lemma 9 to iteratively build a decomposition $D=\left\{D_{1},\ldots ,D_{k}\right\}$ into closed walks where *W* is not a subwalk of any $D_{i}$.

**Case 1.***W* is a path. As such, it does not contain leaving arcs of itself, and by our assumption, leakage(W)>0. Thus, Lemma 9 gives an escaping walk $W_{l}$ (note that the tail of every leaving arc of *W* occurs only once in *W*). Let $w:=\min_{e\in W_{l}}\frac{f\left(e\right)}{{\#}_{W_{l}}\left(e\right)}$, i.e., *w* is the largest weight for which the closed walk $W_{l}$ with weight *w* “fits” into the flow *f*, and add $\left(W_{l},w\right)$ to D. In doing so, we update *f* by subtracting the flow induced by $w\cdot W_{l}$ and remove arcs whose flow value becomes zero (so that each w>0), resulting in a new flow by Lemma 1. We repeat this procedure until some arc e∈W disappears from the graph (i.e., its current flow value becomes 0), and then decompose the remaining flow arbitrarily. If this happens, then no walk of the constructed decomposition contains *W* as subwalk, and thus *W* is unsafe for (G,f).

To conclude, we argue that if no arc of *W* disappears from the graph before $e_{1}$ during our construction, then $e_{1}$ will eventually disappear. Since *W* is a path, both $e_{1}$ and t(l) appear only once in $W_{l}$, and moreover *l* contributes only once in leakage(W). Then, in each iteration we decrease both $f\left(e_{1}\right)$ and leakage(W) by exactly *w*, and since initially $f\left(e_{1}\right)\leq \text{leakage}\left(W\right)$, $f\left(e_{1}\right)$ will eventually become zero.

**Case 2.***W* is a recurring cycle. The main difference with respect to the previous case is that a leaving arc *l* of *W* may contribute in leakage(W) multiple times due to the fact that *W* is a recurring cycle. In fact, if t(l) occurs *q* times as an internal node of *W*, then *l* contributes to the leakage of *W* exactly *q* times. Then, when subtracting the flow induced by $w\cdot W_{l}$, the leakage of *W* will decrease exactly by q·w independently of how long the prefix of *W* is in $W_{l}$ (recall that $W_{l}$ contains only one leaving arc of *W*), whereas the flow of $e_{1}$ will decrease only p·w times, where *p* can be at most the number of times that $t\left(e_{1}\right)$ occurs in *W*. So, even if we build our walk $W_{l}$ using only one leaving arc of *W*, we may still end up by consuming more from leakage(W) than from the flow in $e_{1}$, possibly making the walk have positive excess flow (see Figure 4A for an example of a decomposition built wrongly in this manner). To overcome this, we will escape *W* via the *last* occurrence of a leaving arc of *W*, which will ensure that $f\left(e_{1}\right)$ and leakage(W) decrease equally.

**Figure:**
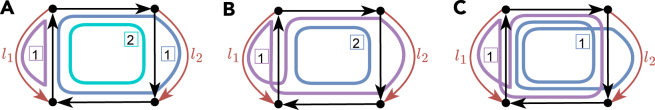
Flow decompositions of an assembly graph Consider the assembly graph shown in Figure 3B and consider the unsafe recurring cycle $W^{\prime }$ obtained by extending the green (safe) walk *W* forward with the arc of flow value 2. Here we show three flow decompositions of the graph, whose walks are in blue, violet, and cyan; their corresponding weights are represented by the boxed numbers. (A) A flow decomposition not avoiding $W^{\prime }$. constructed via an escaping walk for $l_{1}$ (in violet), but *before* the last occurrence of $t\left(l_{1}\right)$ in $W^{\prime }$. (B) A flow decomposition not avoiding $W^{\prime }$. containing one closed walk that uses *both* leaving arcs $l_{1}$ and $l_{2}$. (C) A flow decomposition avoiding $W^{\prime }$. Each leaving arc $l_{i}$ is used in a separate closed walk escaping $W^{\prime }$ via the last occurrence of their tail in $W^{\prime }$.

Since *W* does not contain any of its leaving arcs and leakage(W)>0, we can apply Lemma 9. As such, we obtain an escaping walk $W_{l}$, which we require to traverse *W* until the *last* occurrence of t(l) in *W*.

We proceed as in Case 1 and analogously let $w:=\min_{e\in W_{l}}\frac{f\left(e\right)}{{\#}_{W_{l}}\left(e\right)}$, and add $\left(W_{l},w\right)$ to D until some arc of *W* disappears from the graph (and then decompose the remaining flow arbitrarily).

We again show that during our construction if no arc of *W* disappears from the graph before $e_{1}$, then $e_{1}$ will eventually disappear. Note that in each iteration we decrease both $f\left(e_{1}\right)$ and leakage(W) exactly by ${\#}_{W_{l}}\left(e_{1}\right)\cdot w$, because ${\#}_{W_{l}}\left(e_{1}\right)$ equals the number of times t(l) occurs in *W* (since we took the last occurrence of t(l)∈W) which also equals the number of times *l* contributes in leakage(W), and finally $W_{l}$ contains no other leaving arc of *W*. Since initially $f\left(e_{1}\right)\leq \text{leakage}\left(W\right)$, and we are decreasing some positive value from leakage(W) at every step (since all *w*’s are positive), $f\left(e_{1}\right)$ will eventually become zero. ∎

#### Enumerating safe walks

We dedicate this section to argue the correctness of the following theorem.

##### Theorem 11 (Optimal enumeration of flowtigs)

Given a flow graph (G,f) having n vertices and m arcs, all its maximal flowtigs can be identified in O(||D||)⊆O(mn) time and space, where D is some flow decomposition of total length at most O(mn). The time and space bounds are optimal.

First, we show how to use Theorem 10 to obtain an enumeration algorithm for flowtigs. We achieve this via a standard method also used by e.g. Khan et al.^10^ for the DAG case. However, since a flowtig can also loop around a cycle a number of times depending on the flow values (in the worst case), we additionally show that we can avoid this pseudo-polynomial time complexity with an argument related to the ratio between the flow value of the first arc and the leakage of the cycle. We also show a family of graphs containing Θ(mn) distinct maximal flowtigs, which allows us to conclude that our algorithm is worst-case optimal. Finally, as our algorithm may produce duplicate maximal flowtigs, we also show how to address this using a suffix tree with suffix links, similarly to the approach of Obscura et al..^26^ This increases the runtime of our algorithm to O(||D||logm).

#### Overview

The maximal flowtigs can be identified in O(mn) time by first computing a decomposition D of (G,f) into closed walks and verifying the safety of its subwalks using a two-pointer approach together with our excess flow characterisation. This is correct by definition, since safe walks are subwalks of some closed walk in any flow decomposition.

For the decomposition step, we iteratively find a closed path *P* (which is also a closed walk) and add $\left(P,\min_{e\in P}f\left(e\right)\right)$ to our decomposition until (G,f) is decomposed. This works in O(mn) time and space, since in each iteration we fully decompose at least one out of the *m* arcs of (G,f) and each closed path uses O(n) vertices.

During the two-pointer step, a recurrent operation is that of updating the excess flow of a walk when extending it in the end or the beginning. For that we present a lemma originally presented in Khan et al.^10^ for DAGs, which also holds for our case as excess flow does not depend on the underlying type of graph.

##### Lemma 12 (^10^)

For any walk in a flow graph (G,f), adding an arc e=(u,v) to its start or its end, reduces its excess flow by $f_{in}\left(v\right)-f\left(e\right)$, or $f_{out}\left(u\right)-f\left(e\right)$, respectively. Analogously, removing the arc e=(u,v) from its start or its end, increases its excess flow by $f_{in}\left(v\right)-f\left(e\right)$, or $f_{out}\left(u\right)-f\left(e\right)$, respectively. The quantities $f_{in}\left(v\right)$ and $f_{out}\left(v\right)$ can be computed in O(m+n) time.

#### Two-pointer phase

Based on a decomposition of (G,f), we can compute the maximal flowtigs along each closed walk D∈D using a two-pointer algorithm as follows. We start with the subwalk containing the first arc of *D*. We compute its excess flow *x*, and while x>0 we append the next arc to the walk on the right and incrementally compute its excess flow by Lemma 12. Whenever x≤0, we store the flowtig between the left pointer and the arc preceding the right pointer, and we move the left pointer forward, effectively removing the first arc of the walk, and update the excess flow similarly by Lemma 12. Note that these updates take constant time. We stop when the left pointer returns to the first arc of *D*, implying that we have tried every possible arc in *D* to be the beginning of a flowtig.

#### Ensuring maximality

Note that these flowtigs may only be maximal with respect to the closed walk from where they were scanned. That is, it may happen that a flowtig can be extended using arcs from another closed walk of the decomposition. In any case, to ensure maximality, we check if the walk can be extended to the right and then to the left using arcs of maximum flow. Extending a walk with an arc of maximum flow maximises the excess flow of the extended walk, and thus the unsafety of such an extension implies the unsafety of all other extensions. Therefore, if any extension (left or right) preserves safety, then the walk is not maximal safe. On the other hand, if both extensions individually render the walk unsafe, then the walk is maximal safe. This check can be done in constant time by precomputing a flow-maximum incoming and outgoing arc of every node, which can be done in O(m+n) (recall Lemma 12).

#### Handling recurring cycles

There is an important detail that must be handled in order to achieve the claimed O(mn) runtime, which is in computing safe recurring cycles, i.e. walks along closed walks that repeat arcs as long as their excess flow is positive (note that in Figure 3B we can make the flow values of the inner square arbitrarily large while preserving flow conservation in every node, e.g. by adding a value $k\in Q^{+}$ to every arc of the square; this would make the length of the flowtig drawn in green arbitrarily large). Thus, applying a standard two pointer algorithm would yield an algorithm with only pseudo-polynomial runtime, as the length of such walks depend on the flow values of the arcs. To avoid this, we use the following lemma, together with an argument based on flow conservation.

##### Lemma 13

A safe recurring cycle W with respect to a cycle $C=(v_{1}$, $e_{1},\ldots$, $v_{l}$, $e_{l}$, $v_{1})$ crosses from $e_{l}$ to $e_{1}$ at most $\lceil \frac{f\left(e_{1}\right)}{L}\rceil -1$ times, where L denotes the leakage of the walk $Ce_{1}$.

Proof.

Suppose that the recurring walk *W* consists of c>0 repetitions of the cycle *C*, followed by a non-empty prefix of *C*. If *W* is a cycle, then it does not cross from $e_{k}$ to $e_{1}$. Otherwise, every time *W* walks over $Ce_{1}$ it leaks exactly *L* flow, and so it would leak c·L when doing so *c* times. Since *W* is safe, we have $c\cdot L<f\left(e_{1}\right)\Rightarrow c<\frac{f\left(e_{1}\right)}{L}$. Thus, *c* is at most the greatest integer strictly smaller than $\frac{f\left(e_{1}\right)}{L}$, i.e. $\lceil \frac{f\left(e_{1}\right)}{L}\rceil -1$. ∎

Now we will argue that bootstrapping the two pointer phase with Lemma 13 in the first scanned arc of D∈D is enough to achieve the desired runtime. Clearly, the left pointer moves *n* times, so it remains to analyse the behaviour of the right pointer.

Note that while the left pointer moves from the first to the last arc of *D* it decreases the leakage by at most *L*. Now, if $f_{M}$ and $f_{m}$ denote the maximum and minimum flow occurring in the arcs of *D*, then $f_{M}-f_{m}\leq L$, since to get from $f_{M}$ to $f_{m}$ we need to leak at least *L* units of flow, otherwise $f_{m}$ would not be the minimum flow value occurring in *D*. Then, when the left pointer reaches the last arc of *D* it has introduced at most 2·L of flow to be consumed by the right pointer. Since the right pointer consumes *L* units of flow per complete round around *D* plus the next arc and since the computation of the suffix of the first flowtig uses in the worst case n−1 arcs (after the initial “jump”) we conclude that the right pointer does O(n) transitions in total.

#### Storing walks efficiently

A similar problem to that of computing safe recurring cycles is in their representation since we can not afford to store them explicitly as sequences of arcs. Instead, we use a compact representation inspired by Khan et al..^10^ Let $D=\left\{D_{1},\ldots ,D_{k}\right\}$ be a flow decomposition and let *W* be a flowtig. Then, we represent *W* by three integers (i,j,l), where *i* points to the closed walk $D_{i}\in D$ where *W* occurs, *j* points to the first arc of *W* in $D_{i}$, and *l* denotes the length of *W*. This encoding allows us to uniquely identify flowtigs with additional constant space per flowtig.

#### Optimal enumeration of flowtigs

In Figure 5 we present a family of graphs requiring Ω(mn) total space to decompose its flow and containing Θ(mn) distinct maximal flowtigs. This structure is identical to the one presented in,^10^ except that we add an arc from $b_{k}$ to $a_{1}$ to make the graph into a circulation.

**Figure:**
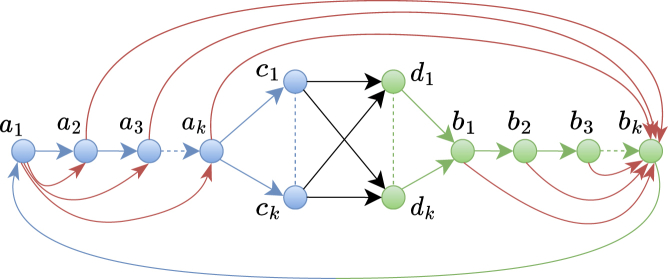
Worst-case class of graphs for flow decompositions and flowtigs A family of graphs parameterized by k≥1 with Θ(k) nodes, at least Θ(k) arcs (sparsest case) and at most $\Theta \left(k^{2}\right)$ arcs (densest case). The black arcs have *k* flow, the red arcs have unit flow, and the remaining arcs are according to flow conservation. Such flow graphs contain Θ(mn) unique maximal flowtigs, showing that our algorithm is optimal.

About the structure of the graph, observe that by choosing k=n/4 and any subset of connections between $C=\left\{c_{1},\ldots ,c_{k}\right\}$ and $D=\left\{d_{1},\ldots ,d_{k}\right\}$ that make the graph strongly connected, we get a graph with any *n* and *m*. Let there be flow *k* on the black arcs and unit flow on the red arcs. The remaining arcs are according to flow conservation. Further, note that m=Θ(|C×D|) and that n=Θ(k).

For the analysis of the flow decomposition D, note that each arc in C×D necessarily contributes in D with at least one closed walk of length *at least* Θ(k), implying that ||D||=Ω(mn).

For the analysis of the maximal flowtigs, observe that there are |C×D| distinct flowtigs from $a_{i}$ to $b_{i}$ for all 1≤i≤k because every path from $a_{i}$ to $b_{1}$ has excess flow *i*. For all 2≤j≤k−1, the $a_{j}$-$b_{j}$ flowtigs are maximal, and the $a_{1}$-$b_{1}$ and $a_{k}$-$b_{k}$ flowtigs can only be extended by adding the green and blue arc to the beginning and end, respectively. Therefore, we have Ω(|C×D|·k)=Ω(mn) distinct maximal flowtigs.

To conclude our analysis, recall that for any flow graph there is a O(mn)-sized flow decomposition, and so the graph shown in Figure 5 only admits flow decompositions of size Θ(mn). Moreover, the *number* of distinct maximal flowtigs is bounded from above by the size of the smallest flow decomposition in the underlying graph. Indeed, this is true by definition of safety, and due to the fact that in any closed walk D∈D we have at most one unique maximal flowtig per arc in *D*. Then, the class of graphs shown in Figure 5 have Θ(mn) distinct maximal flowtigs, as we wanted. This shows that our algorithm is worst-case optimal in time and space.

#### Deduplication

Finally, our algorithm may produce duplicate maximal flowtigs. To address this we use a suffix tree with suffix links to identify the set of maximal flowtigs without duplicates, similar to Obscura et al.^26^ This increases the runtime of our algorithm to $O\left(\left|\left|D_{f}\right|\right|\log\;m\right)$.

A maximal flowtig may be reported multiple times when there exist distinct $D_{i},D_{j}\in D$ such that $D_{i}\cap D_{j}\neq \emptyset$. However, maximal flowtigs that repeat any arc cannot be reported multiple times, as that would imply that there exist two equivalent cycles in the decomposition, which can not happen in our algorithm. Hence, we only need to deduplicate paths and cycles.

For filtering out duplicates efficiently, we can apply an idea similar to that of Obscura et al..^26^ We build a string $S:=D_{1}D_{1}D_{1}D_{1}D_{1}D_{1}\#D_{2}D_{2}D_{2}D_{2}D_{2}D_{2}\#\cdots \#D_{k}D_{k}D_{k}D_{k}D_{k}D_{k}$ of length $O\left(\left|\left|D_{f}\right|\right|\right)$ for our decomposition $\left\{D_{1},\ldots ,D_{k}\right\}$ which contains the arcs of our decomposing cycles. Then we build a suffix tree in $O\left(\left|\left|D_{f}\right|\right|\right)$ time using an algorithm by Farach,^42^ which we can apply since our nodes form an integer alphabet. Then we add suffix links into the tree using a linear-time algorithm by Maaß.^43^ Suffix links are arcs that point from a string $a_{1}\cdots a_{l}$ to a string $a_{2}\cdots a_{l}$.

Now, we can walk along the suffix tree while executing the two-pointer algorithm. After finding the initial walk in the two-pointer algorithm, we check if the initial walk completes the decomposing cycle at least twice. If it does, then all maximal flowtigs reported by the two-pointer algorithm will repeat at least one arc, and hence cannot have duplicates. In this case, we do not need to use the suffix tree.

If on the other hand the initial walk completes the decomposing cycle less than two times, we make use of the suffix tree. We first traverse the suffix tree from the root to find the initial walk, and then traverse one step deeper for each move of the right pointer, and use a suffix link for each move of the left pointer. Since the right pointer moves at most three times around the cycle, and the initial walk repeats the cycle less than two times, repeating each decomposing cycle six times in the string *S* is enough for never entering a leaf when moving the right pointer.

Using a suffix link takes constant time. However, when stepping deeper into the tree, we have up to O(m) options, of which only one is correct. To choose the right one we can use binary search which takes O(logm) time. Hence, while executing the two-pointer algorithm, we can traverse the suffix tree in constant time for each left pointer update, and in O(logm) for each right pointer update. In total this takes $O\left(\left|\left|D_{f}\right|\right|\log\;m\right)$ time for all executions of the two-pointer algorithm.

Using the simultaneous traversal of the suffix tree, we can mark nodes of the suffix tree whenever we output a maximal flowtig. Then, before we output a maximal flowtig, we check if the corresponding node was marked already, and only output it if the node is unmarked.

#### Applying flowtigs in practice

In this section we describe a proof-of-concept application of flowtigs on real data. This is based on the fact that the definition of excess flow does not require flow conservation to hold. As such, on real graphs where the coverage values of the arcs do not satisfy flow conservation, we can still define a walk *W* to be a flowtig if it has positive excess flow. However, the lack of flow conservation makes enumerating flowtigs harder, because graphs without flow conservation do not admit flow decompositions, and thus we cannot apply a two-pointer style algorithm over each closed walk of the decomposition like we did under the assumption of perfect data.

We show how to enumerate all maximal flowtigs in graphs built from real data and describe two heuristics to filter such flowtigs in order to improve their accuracy. Finally, we show experimental results of flowtigs applied to real data.

#### Enumerating flowtigs without flow conservation

Corollary 7 does not hold in graphs without flow conservation in every node, and so, in general, any type of walk can be safe. Consequently, we cannot know beforehand which type of walks to look for. Moreover, it may happen that a closed walk does not have any leaving arcs, which makes the walk safe and of infinite length according to definition. For example, consider an arc *e* followed by a cycle *C* that does not contain any leaving arcs. Then, the walk eC…C is safe, where *C* can be repeated arbitrarily often.

Our algorithm (Algorithm 1) essentially performs an exhaustive search from each arc in a depth-first search fashion, hence finding all the flowtigs starting with that arc. It keeps extending the current flowtig using the excess flow characterization as a heuristic. If at any point the current flowtig does not admit safe extensions, then we can stop the search on that branch since no further extension will make it safe (see Figure 6 for an illustration). Note that we maintain the invariant that the extension subroutine is called only with flowtigs, and thus only flowtigs can be added to the output set. To handle infinitely long safe walks, observe that a walk has no leaving arcs if and only if it has zero leakage, and therefore its excess flow is simply the flow on the first arc of the walk. Then, whenever we are performing an extension with an arc that is already part of the current flowtig but the current excess flow equals the excess flow at the time we first visited the revisited arc, we can correctly abandon this branch. Finally, whenever a given flowtig does not admit any safe extensions, we test it for maximality. Observe that any maximal flowtig contains a first arc, and since we perform an exhaustive search starting in every arc of *G*, we will eventually compute all the maximal flowtigs of *G*.

Algorithm 1 **Flowtigs - exhaustive output-sensitive search**

**Function** extend *(W, S)* **:**

 
$W:=\left(v_{1},e_{1},\ldots ,v_{t-1},e_{t-1},v_{t}\right)$

**foreach** *arc* $e=\left(v_{t},x\right)$ *where* $x\in N^{+}\left(v_{t}\right)$ **do**

$W^{\prime }:=\left(v_{1},e_{1},\ldots ,v_{t-1},e_{t-1},v_{t},e,x\right)$

**if** e∈W *and* $\text{excess}\left(W^{\prime }\right)=\text{excess}\left(e-prefix\;of\;W\right)$ **then**

**break**

end

**if** $W^{\prime }$ *is a flowtig* **then**

**extend**($W^{\prime },S$)

end

end

 
**if** *W is a maximal flowtig* **then**

S:=S∪{W}

end

**return**

S:=∅

**foreach** *arc* e=(u,v) *of G* **do**

extend((u,e,v),S)

end

**return** *S*

**Figure:**
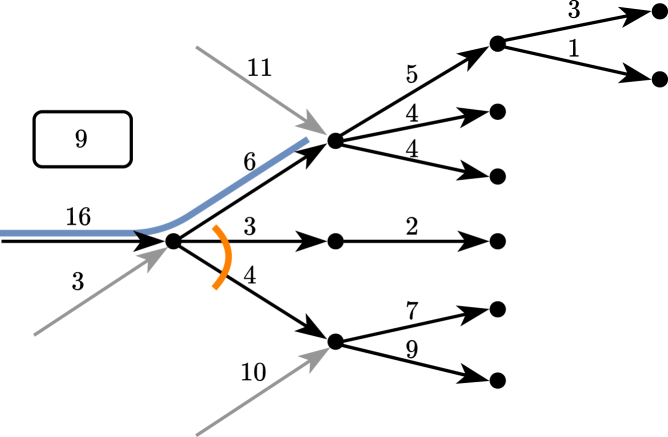
Execution of the practical flowtigs algorithm The current flowtig is represented in blue. The leakage when extending from the arc with flow 16 to the arc with flow 6 is highlighted in orange. The arc with flow 16 is also the first arc of the current flowtigs search. In the boxed number we store the current excess flow. Assuming T=0, the blue walk admits one safe right extension only, which is through the arc with flow 5.

Algorithm 1 runs in Θ(m·out) time, where out denotes the total length of all the flowtigs (including all non-maximal ones). This bound follows from the fact that any branch of the recursion starting on a given arc takes time proportional to the length of the flowtig it computes and any given branch of the recursion spends at most *m* time to reject its unsafe extensions (recall that we are working with multigraphs). Note that the longest length of a flowtig is O(mU/u), where *U* denotes the largest flow value and *u* the smallest flow value in the arcs of *G* and $U,u\in Q^{+}$. Further, a single arc *e* can be safely extended to the right in at most *m* ways, and any such extended walk admits, again, at most *m* safe extensions. Following this argument, we can see that the total length of the right-maximal flowtigs starting at *e* is $O\left(m^{mU/u}\right)$, which allows us to conclude that $out=O\left(m^{1+mU/u}\right)$. From this discussion we get the following theorem.

##### Theorem 14 (Enumeration of flowtigs in practice)

Given an assembly graph built from real data, it is possible to find all its flowtigs in $O\left(m^{2+mU/u}\right)$ time, where U denotes the largest flow value and u the smallest flow value in the arcs of G.

#### Filtering flowtigs

Excess flow can be interpreted as the amount of flow that is guaranteed to pass contiguously through the flowtig. With varying coverage values, instead of reporting all flowtigs with any excess flow greater than 0, we can instead report all flowtigs with excess flow greater than a given threshold *T*. In practice, on e.g. de Bruijn graphs, such a threshold could be derived as function of the minimum *k*-mer abundance required when building the graph. As such, we introduce the following definition.

##### Definition 15 (*T*-flowtig)

A walk W is a T-flowtig if excess(W)>T. A T-flowtig is maximal if it is not a subwalk of another T-flowtig.

As another refinement, we can change the maximality check so that we do not report a maximal *T*-flowtig if and only if it is a prefix of some $T^{\prime }$-flowtig with $T^{\prime }\leq T$. Indeed, we observed that dropping some maximal *T*-flowtigs according to this filter decreases the number of misassemblies while maintaining the values of other metrics. For this matter, we introduce the following definition.

##### Definition 16($\left(T^{\prime },T\right)$-flowtig)

A walk W is a $\left(T^{\prime },T\right)$-flowtig if it is a T-flowtig and it is not a prefix of any $T^{\prime }$-flowtig.

Thus, a *T*-flowtig $W=\left(v_{1},e_{1},\ldots ,v_{l-1},e_{l-1},v_{l}\right)$ is reported if it satisfies two properties: i) excess(W)>T, and ii) $\text{excess}\left(W^{\prime }\right)\leq T^{\prime }\leq T$ where $W^{\prime }=We_{l}$ and $e_{l}=\left(v_{l},v_{l+1}\right)$. This implies that this extension of *W* leaks at least $\text{excess}\left(W\right)-T^{\prime }$, since $\text{excess}\left(W^{\prime }\right)\leq T^{\prime }\Leftrightarrow f_{out}\left(v_{l}\right)-f\left(e\right)\geq \text{excess}\left(W\right)-T^{\prime }$, and the quantity $f_{out}\left(v_{l}\right)-f\left(e_{l}\right)$ is exactly the increment in leakage when extending *W* to $W^{\prime }$.

#### Experiments on simulated data

We compare flowtigs against unitigs, extended contigs and structural contigs on various datasets. As this work primarily focuses on improving assembly contiguity, for storage and deduplication of the assembly sequences we exploited standard Rust libraries, which may not be optimal in terms memory consumption. Although the choice of such string processing methods has no effect on the assembly contiguity, for a more efficient practical implementation one may use more optimised string algorithms and libraries. Additionally, we handle the bidirectedness of genomic data using doubled graphs.^44^ This allows us to employ our flowtig definition as it is, but results in contigs being output twice, once forwards and once in reverse. We filter the contigs as postprocessing to output only the lexicographically smaller direction.

Since flowtigs overlap, we cannot use standard metrics for contiguity such as NGA50, because they get artificially inflated through overlaps. Instead, we use the EA50 family of metrics, which is an improvement over NGA50 that is robust against overlapping contigs.^35^ The EA50 is computed by aligning the contigs to the reference, and for each reference base identifying the longest contig that aligns to it. These lengths are then sorted and for e.g. EA50, the 50-percentile is reported. EA75 works analogously, by reporting the 75-percentile of largest values. For non-overlapping contigs NGA*x* is equal to EA*x* for all *x*.

In our experiments with simulated data we aim for a very precise evaluation. However, our evaluation software QUAST^45^ is not compatible with our formulation of flowtigs, as it does not allow a contig to repeat a genome. It would treat these cases as misassemblies, resulting in an inaccurate computation of the EA*x* metrics. Therefore, we forbid a subwalk *X* from repeating arcs of an underlying closed walk *W* for our experiments on simulated data. The corresponding theory results can be obtained by adding only minor restrictions to the more general results. More specifically, any flowtig that repeats no arc is safe, because if it is a subwalk of each flow decomposition where a subwalk is allowed to repeat arcs, then it is also a subwalk of each flow decomposition where a subwalk is not allowed to repeat arcs. Therefore, we only report safe walks.

##### Datasets

See Table 1 for an overview of the metagenomic datasets we use for our experiments. The datasets *simple7*, *medium20* and *complex32* are from Shafranskaya et al.,^22^ containing 7, 20 and 32 bacterial genomes, across 6, 19 and 26 genera, respectively. We selected these datasets to be able to see the effects of flowtigs on bacterial communities with various complexities. We refer the reader to^22^ for more details on these three datasets. Additionally, we also include the mock community *JGI*^20^ which contains 23 bacterial and 3 archaeal strains with finished genomes, which we select as a microbiome that does not only contain bacteria. For more realistic datasets, we select *HMP*,^21^ which contains 23 high-quality assembled genomes of a real waste water sample. Since the datasets JGI and HMP are missing abundance profiles, we simulate them using the log-normal distribution. This is used by various state-of-the-art metagenomic read simulators.^46^^,^^47^ We parameterise the log-normal distribution with a mean of 0 and a standard deviation of 2, which results in a realistic abundance profile for e.g. the human gut.^19^ To the best of our knowledge, JGI and HMP are not motivated by specific real metagenomes with available abundance profiles, hence we use this parameterisation to simulate their abundance profiles. To get integer abundances, we round the output of the log-normal distribution, and, for reproducibility, we fix the seed for the random generator. The datasets used for our experiments are available on Zenodo.^36^

##### Comparing assembly contiguity

In the EA50 and EA75 rows of Table 2 we show in parentheses the relative improvement with respect to unitigs. In this way we can see that flowtigs give a notable improvement in comparison to structural contigs.

As we can see, flowtigs provide consistently better EA50 and EA75 values than unitigs on all datasets (13%–17% better on EA50 and 14.4%–100.4% better on EA75). Moreover, our improvements over unitigs are larger on shorter contigs (EA75) than longer contigs (EA50), with the exception of HMP where these values are still very close. On the other hand, structural contigs provide only minimal improvements over unitigs: up to 2.17% for EA50 on all datasets, and up to 4.56% for EA75, on all datasets, excluding complex32. On complex32, under EA75 all contigs provide significant improvements over unitigs, which might be due to the fact that the EA75 value of unitigs is already very small (e.g. compared to the other four datasets). That is, the graph of complex32 has mostly short unitigs (i.e., to cover 75% of the genomic position one needs unitigs of length at least 538) due to branching arising from the many species in the graph; extended contigs, structural contigs and flowtigs are thus likely to be extended over such branching nodes. Furthermore, the improvement that flowtigs bring over unitigs is consistently several times higher (between 4 × for EA75 in complex32 and 14 × for EA50 in HMP) than what structural contigs provide over unitigs. Exceptionally, in the simple7 dataset we have an improvement of 123 ×, which is due to the fact that the improvement of structural contigs over unitigs is of just 0.105%.

In Table 2 we also include the genome fraction in our table even though in theory it should be 100% in each case since we add unitigs and work on error-free data. In practice it is slightly lower, since QUAST uses approximate alignment that does not align very short contigs. If it was much lower than 100%, this may be an indicator that our EA*x* statistics are skewed, as they are based on alignments. However, it is above 99% in all but one cases and only very short contigs are unaligned, specifically all unaligned contigs are shorter than 150bp in all datasets. For the unitigs of complex32, we can assume that they contain many very short contigs, as the graph is very complex, which explains why the genome fraction is only 94% in this case. The EA*x* metrics are based on the longest contigs, similar to the well-known NGA*x* metrics. Hence, we take the genome fraction as an indicator that our statistics are accurate.

To sum up, flowtigs provide the first notable improvement over both long unitigs (EA50) and short unitigs (EA75) under the assumption of perfect data. At the same time, they admit a simpler characterisation than structural contigs (i.e. excess flow).

##### Comparing performance

We also compare the performance of the various algorithms in Table 3. Note that unitigs are computed with BCALM2,^31^ a highly engineered parallel external memory algorithm, while all other algorithms run single-threaded with a less engineered proof-of-concept implementation. Further, unitigs are the input to all other algorithms, but we report the runtimes of the other algorithms without that of unitigs. In a practical assembler, the graph will likely be computed with a different tool than BCALM2, hence we get a better comparison between the algorithms if we ignore the initial graph-building step.

The algorithm for extended contigs is very simple and runs in linear time, which is visible in its great performance compared to structural contigs and flowtigs. Structural contigs are computed with an O(mn) algorithm, which becomes notable on the larger graphs of complex32 and JGI, where the runtime is significantly higher than that of the other algorithms. But while flowtigs are computed with an $O\left(\left|\left|D_{f}\right|\right|\right)$ algorithm which is O(mn) in the worst case as well, the actual size of the flow decomposition in our graphs is much lower than that as shown in Table 1. Hence, it runs much faster and behaves more like the linear-time extended contig algorithm than the quadratic structural contig algorithm.

Regarding memory usage, we see that extended contigs and structural contigs are very similar, while flowtigs use roughly an order of magnitude more memory, up to almost 4GiB. This is because extended contigs and structural contigs use compact data structures to store strings and do not require deduplication, while our flowtigs implementation uses simpler methods to store strings as well as a simple method for deduplication. We assume that using a more compact method to store strings and an optimised way to do deduplication would reduce the RAM usage of flowtigs down to around that of structural contigs.

#### Experiments on real data

We now compare the performance of flowtigs against other contigs on real data. Note that we are unable to use the O(mn)-time two-pointer algorithm (recall Theorem 11) since it assumes flow conservation in every node. Therefore, in this section we use Algorithm 1. Note that this algorithm works with both perfect and real data (whereas the O(mn)-time two-pointer algorithm works only on simulated data), but has the downside that its running time is worse. Note that for real data we do not need to change the definition of flowtigs as we did for simulated data. While QUAST does not support our original definition of flowtigs it still reports a very low number of misassemblies with the original definition of flowtigs, and hence we deem the original version as better in practice.

##### Dataset

Our experiments with real data were performed on the ZymoBIOMICS Gut Microbiome Standard dataset^48^ (henceforth referred to by Zymo), a microbial community that mimics the human gut microbiome. It features 21 strains of 17 species, and, as highlighted in,^17^ it includes five strains of Escherichia coli at 8% abundance each (see Table 1 for other features of the dataset). As the input to our algorithm, we use a library of PacBio HiFi reads of this community, available in the sequence read archive^49^ under the accession SRR13128014.

##### Assembly contiguity

Similarly to the table showing our results in the case of simulated data, we also show in parentheses in the EAx rows of Table 4 the relative improvement with respect to unitigs.

As we can see, flowtigs provide consistently better EA values than unitigs and extended contigs on all datasets. In particular, our improvement over unitigs is up to one order of magnitude larger than the improvement of extended contigs over unitigs. For 0-flowtigs, although we get substantial improvements over all EA metrics, we also have up to 4 times more misassemblies than unitigs and up to 3 times more misassemblies than extended contigs. This undesired consequence of getting more misassemblies when attempting to find longer contigs is mitigated by applying the concept of *T*-flowtig for T≠0. For example, going from 0-flowtigs to 7-flowtigs, we observe that the EA metrics remain essentially unaltered while the number of misassemblies drastically reduces. This trend is even more noticeable when we apply the filtering technique to 7-flowtigs (see the filtering flowtigs section in STAR Methods). Indeed, with (0,7)-flowtigs, the EA metrics are not as high to that of (unfiltered) 7-flowtigs, but still are around one order of magnitude higher than extended contigs are with respect to unitigs, and, moreover, the number of misassemblies reduces to less than half to that of extended contigs. To sum up, by applying the concept of $\left(T^{\prime },T\right)$-flowtigs we are able to achieve substantially better contiguity while decreasing the number of misassemblies and losing on genome fraction just slightly. We pushed this idea further and tested 10-flowtigs. Note that the impact of our threshold is easy to see: for $T^{\prime }>T$, the $T^{\prime }$-flowtigs are a subset of the *T*-flowtigs, and thus the number of misassemblies as well as EA metrics do not increase (left to right in the order of the columns corresponds to imposing more restrictions on the reported contigs). Unsurprisingly, our experimental results show precisely that, and further show that choosing a value for *T* can have a great impact on the metrics presented. For example, in 10-flowtigs we get lower EA metrics (e.g., for EA50 we get a value of 0). This happens since in practice we are excluding too many flowtigs from the set of (0,7)-flowtigs, and naturally we also get a very low number of misassemblies for every value of *k*. This allows us to conclude that the chosen value of 10 for 10-flowtigs is too high for this dataset.

##### Performance

We show in Table 5 the time and memory consumption of Algorithm 1 in the Zymo dataset for different values of *k*. Again, unitigs are computed with BCALM2,^31^ and we report the runtimes of the other algorithms without that of unitigs. The algorithm for extended contigs is the same as the one described in the case of simulated data.

Due to its simplicity, the algorithm for extended contigs has a much better performance than Algorithm 1 both in time and space. This was expected as the extended contigs algorithm is a linear time simple algorithm requiring minimal data structures to function, whereas our real-data flowtigs algorithm essentially performs an exhaustive search on the graph. It is more interesting to analyse the performance between different types of flowtigs. We observe that increasing the threshold of the flowtigs decreases the memory usage, which was expected since the search on a given branch of the search is pruned earlier for higher values of *T*. For the same reason, for increasing values of *T* the execution time decreases. With respect to execution time, this trend is not perfect experimentally, as it can be observed for example in the case where k=31 between 0-flowtigs and 7-flowtigs, but it holds almost everywhere. Note that including the filtering in the algorithm does not require more time nor space. Finally, observe that for increasing values of *k* the time and memory requirements decrease simply because the DBGs are smaller, but on the negative side, as one can see in Table 4, the improvement in the contiguity metrics with respect to unitigs decreases for higher values of *k*.

#### Details about implementation and evaluation

We implement the flowtig algorithms for simulated and real data in Rust. Instead of the complex deduplication algorithm we use a hash set to deduplicate the output. The implementation is available on github^37^ and on Software Heritage^38^ under the BSD-2-Clause license. Our experiment pipeline is written with snakemake^50^ and available on Software Heritage^51^ under the BSD-2-Clause license. To compare against extended and structural contigs, we use the implementation by Schmidt available on Software Heritage^52^ under the BSD-2-Clause license.

The algorithms for extended contigs and structural contigs use a compact representation of the input strings, storing all strings 2-bit encoded in a single bitvector. Further, they never make any copies of these strings, and hence use only a low amount of memory. Compared to that, our flowtig implementations store strings in ASCII format. During deduplication, we copy the unitigs into the flowtigs and store them inside a hash set, hence it holds the input strings and all flowtigs simultaneously in memory. This causes it to use around an order of magnitude more memory. If we were to use the compact string format for flowtigs as well and would deduplicate while storing the flowtigs as sequences of arcs without copying the strings, then our memory usage would likely be similar to that of structural contigs.

When computing longer safe walks, we get an issue with a lower genome fraction that was already noticed by Jain^53^ on overlap graphs. By only using maximal contigs, some genomes may be left with a gap where no contig aligns, even on perfect data. See for example Figure 1, where in (C) the middle horizontal arc is not covered by any contig that aligns to the red genome, and in (D) there is no contig that aligns to the blue genome outside of the repeat. To mitigate this effect, we always run our evaluations on the maximal contigs combined with unitigs. For example with flowtigs on complex32, the genome fraction would only be 99.29% without adding unitigs, while it is 99.77% with adding unitigs. See the table below for the genome fractions of flowtigs on all datasets with and without adding unitigs.

**Table:** Genome fraction of flowtigs without and with unitigs

Dataset	only flowtigs	flowtigs+unitigs
simple7	99.96	99.99
medium20	99.96	99.98
complex32	99.29	99.77
JGI	99.94	99.97
HMP	99.92	99.95
Zymo	54.94	66.41

For the Zymo dataset we used our algorithm suited for real data with T=0 (Algorithm 1).

We compare flowtigs against unitigs, extended contigs, and structural contigs. On simulated data, for each metagenomic reference, we circularise each genome, and then compute a compacted de Bruijn graph for with *k*-mer abundances for k=31 using bcalm2.^31^ Since bcalm2 does not support setting a multiplier for each genome, we simply feed bcalm2 *a* copies for a genome with abundance *a*. The sizes of the de Bruijn graphs are displayed in Table 1. From the compacted de Bruijn graph we then compute the various tigs. We evaluate the tigs with QUAST^45^ using various common metrics. Since we use error-free data, we do not use metaQUAST,^54^ because its changes over QUAST are only regarding erroneous data. In addition to QUAST’s metrics, because flowtigs can overlap a lot, we add the EA50 family of metrics, which is an improvement over NGA50 that is robust against overlapping contigs.^35^ The EA50 is computed by aligning the contigs to the reference, and for each reference base identifying the longest contig that aligns to it. These lengths are then sorted and for e.g. EA50, the 50-percentile is reported. EA75 works analogously, by reporting the 75-percentile of largest values. We implement this metric in a modified version of QUAST which additionally computes average contig lengths and does not filter out short contigs. It is available on Software Heritage^55^ under QUAST’s original custom license.

Note that QUAST filters alignments below a length of 65 regardless of its parameterisation, hence very short contigs do not align in our evaluation, and we get a genome fraction below 100% even though we work with perfect data and safe algorithms only.

#### Instructions for reproducing our experiments

We run our experiments on a snakemake (^50^) pipeline.^51^ To reproduce our experiments with simulated data, clone the pipeline from github with git clone https://github.com/elieling/safe-paths-with-flowtigs.git and download Anaconda.^56^ In the project directory, create the required environment with conda env create environment.yml, then activate the environment with conda activate snakemake-flowtigs.

#### Reproducing experiments with simulated data

First, ensure you are using the correct version by running in the project directory git checkout a2698923762b58129fd027602f242209cecdd2d7.

Then, include the reference of the desired metagenome in the folder safe-paths-with-flowtigs/data/meta/<name_of_the_dataset>.

The folder should contain a file ending in .fna or .fasta for each genome in the reference metagenome, as well as a file named nanosim.abundances.tsv containing the abundance information in tsv format, so that the first column contains the name of the genome and the second column contains its abundance in percentage. If the abundance file is missing, abundances will be simulated. Then, the file safe-paths-with-flowtigs/data/meta/<name_of_the_dataset> should be copied to safe-paths-with-flowtigs/data/meta/<name_of_the_dataset>_reference, because we also simulate the reads when working with simulated data. Then, the pipeline is run with the command snakemake --use-conda -j 1 "<path>/safe-paths-with-flowtigs/data/reports/output/ meta_<metagenome>_k<k>ma1t<threads>nm1/<name_of_the_report>/report.tex".

The output report will be located in the file data/reports/output/meta_<metagenome>_k<k>ma1t<threads>nm1/ <name_of_the_report>/report.tex.

#### Reproducing experiments with real data

To ensure you are using the correct version, run in the project directory git checkout bd04d0789f313415293ac3bdace399c6b0c035e5.

Then, include the desired reads in the file safe-paths-with-flowtigs/data/meta/<name_of_the_dataset>/reads.fq and the reference of the desired metagenome in the folder safe-paths-with-flowtigs/data/meta/<name_of_the_dataset>_reference.

The reference metagenome should be in the same format as with simulated data, but does not need an abundance file. In our experiments, we use the Zymo reads with accession SRR13128014 and we use a minimum abundance threshold of 5, i.e., we exclude all edges that have a multiplicity lower than 5. Then, the pipeline is run with the command snakemake --use-conda -j 1 "<path>/safe-paths-with-flowtigs/data/reports/output/ real_<metagenome>_k<k>ma<minimum_abundance>t<threads>nm0th<threshold>/

<name_of_the_report>/report.tex".

The output report will be located in the file data/reports/output/ meta_<metagenome>_k<k>ma<minimum_abundance>t<threads>nm0th<threshold>/ <name_of_the_report>/report.tex.

To run the pipeline without using the additional filtering, instead run snakemake --use-conda -j 1 "<path>/safe-paths-with-flowtigs/ data/reports/output_no_filtering/

real_<metagenome>_k<k>ma<minimum_abundance>t<threads>nm0th<threshold>/

<name_of_the_report>/report.tex".

The output report will then be located in the file

data/reports/output_no_filtering/

meta_<metagenome>_k<k>ma<minimum_abundance>t<threads>nm0th<threshold>/<name_of_the_report>/report.tex.
